# Improving Ethical and Participatory Practice for Marginalized Populations in Biomedical HIV Prevention Trials: Lessons from Thailand

**DOI:** 10.1371/journal.pone.0100058

**Published:** 2014-06-20

**Authors:** Dan Allman, Melissa Hope Ditmore, Karyn Kaplan

**Affiliations:** 1 Dalla Lana School of Public Health, University of Toronto, Toronto, Ontario, Canada; 2 The CIHR Social Research Centre in HIV Prevention (SRC), Dalla Lana School of Public Health, University of Toronto, Toronto, Ontario, Canada; 3 Sex Workers Project, Urban Justice Center, New York, New York, United States of America; 4 Thai AIDS Treatment Action Group (TTAG), Bangkok, Thailand; The George Washington University Medical Center, United States of America

## Abstract

**Background:**

This paper presents findings from a qualitative investigation of ethical and participatory issues related to the conduct of biomedical HIV prevention trials among marginalized populations in Thailand. This research was deemed important to conduct, as several large-scale biomedical HIV prevention trials among marginalized populations had closed prematurely in other countries, and a better understanding of how to prevent similar trial closures from occurring in the future was desired.

**Methods:**

In-depth key informant interviews were held in Bangkok and Chiang Mai, Thailand. Interviews were audio recorded, transcribed, translated and thematically analyzed. The Good Participatory Practice Guidelines for Biomedical HIV Prevention Trials (GPP) guided this work.

**Results:**

Fourteen interviews were conducted: 10 with policymakers, academic and community-based researchers and trial staff and four with representatives of non-governmental organizations (NGOs). Suggested ways to improve ethical and participatory practice centered on standards of HIV prevention, informed consent, communication and human rights. In particular, the need to overcome language and literacy differences was identified. Key informants felt communication was the basis of ethical understanding and trust within biomedical HIV prevention trial contexts, and thus fundamental to trial participants' ability to exercise free will.

**Discussion:**

Biomedical HIV prevention trials present opportunities for inclusive and productive ethical and participatory practice. Key informants suggested that efforts to improve practice could result in better relationships between research stakeholders and research investigative teams and by extension, better, more ethical participatory trials. This research took place in Thailand and its findings apply primarily to Thailand. However, given the universality of many ethical considerations, the results of this study can inform the improvement of ethical and participatory practice in other parts of the world where biomedical HIV prevention trials occur, and where clinical trials in marginalized populations continue.

## Background

This paper aims to contribute to efforts to improve ethical and participatory practice for marginalized populations in biomedical HIV prevention trials. The focus of this paper is Thailand. However, owing to the universality of many ethical principles, the results presented here can inform the improvement of ethical and participatory practice in other parts of the world where biomedical HIV prevention and other clinical trials seek to involve marginalized populations.

In the 2000s, several large-scale biomedical HIV prevention trials among marginalized populations prematurely closed in a number of countries in Africa and Asia. These trials sought to test the efficacy of Tenofovir (Viread) as pre-exposure prophylaxis (PrEP) for HIV prevention in locations as diverse as Thailand, Cambodia, Malawi, Nigeria, Ghana and Cameroon [1]. The closure of these trials acted to prevent or in some cases slow the development of other biomedical HIV prevention trials in marginalized populations across these regions. The reasons for these closures have been documented at length [1]–[5]. The role of ethical and participatory practice in these trial stoppages has received substantial attention also [6]–[10].

The research described in this paper was undertaken to understand how to prevent similar trial closures from occurring in the future. The research was conducted in Thailand as Thailand had been one of the original sites of the planned PrEP trials, and because Thailand has been the site of many trials and the focus of previous efforts to understand and improve applied ethics within biomedical HIV prevention trial contexts [11]–[13].

*The Good Participatory Practice Guidelines for Biomedical HIV Prevention Trials* (GPP) guided the work reported here. UNAIDS (the Joint United Nations Programme on HIV/AIDS) and AVAC (Global Advocacy for HIV Prevention) developed the GPP Guidelines in the years following the trial closures noted above. The first edition of the GPP Guidelines was published in 2007 [14], and a revised second edition in 2011 [15]. The GPP Guidelines include guidance related to various ethical principles. The guidelines offer advice to trial practitioners in order to promote participatory practice. A central intent of participatory practice is to address some of the concerns that led to the premature trial closures noted above.

In the seven years since its initial publication, the GPP Guidelines have had substantial impact. They have been widely discussed, promoted and endorsed within editorial and commentary [8], [16]–[21], in terms of ethical practice [7], [22]–[39], and in terms of best practice [40]–[60]. They have been considered within the context of HIV prevention social science research [5], [9], [61]–[73], pragmatically applied within biomedical HIV prevention trials [74]–[78], and noted in the U.S. Presidential Commission for the Study of Bioethical Issues under the Obama Administration [79]. In addition, the GPP Guidelines have influenced developments outside the field of HIV, notably within research on malaria and tuberculosis [80]–[85].

The GPP Guidelines have been translated into multiple languages, including Thai [86]. The present research gathered and analyzed qualitative data from a diversity of key informants from stakeholder groups in Thailand. The goal of the research was to show stakeholder perspectives concerning barriers and improvements to ethical conduct and community engagement.

## Methods

### Ethics Statement

The HIV Research Ethics Board under the Office of Research Ethics at the University of Toronto approved the research reported here (Protocol Reference # 25708). Informed consent from all research participants was obtained as per the guidelines of the aforementioned Research Ethics Board.

### Methods

This qualitative research project collected recommendations from people involved at a variety of levels with biomedical HIV prevention trials in Thailand. It accomplished this through the utilization of key informant interviews. Four categories of stakeholders were involved: i) academic researchers and research site staff, ii) policymakers, iii) staff of community-based organizations and non-governmental organizations (NGOs), and iv) community-based researchers.

Utilizing purposive sampling, key informants with high levels of expertise in their respective fields were recruited. Criteria for recruitment included individuals who would have an understanding of existing or previous biomedical HIV prevention trials in Thailand, the challenges of involving marginalized populations within such studies, and areas deemed important to trial conduct as highlighted by the GPP Guidelines. Key informants were offered Thai Baht 500 (approximately 16 USD) as compensation for their time and Thai Baht 200 (approximately 6 USD) reimbursement for their travel expenses. Key informants from NGO, academic and government posts waived any compensation or reimbursement.

In total, 14 in-depth interviews were held in Bangkok and Chiang Mai, Thailand. Two of the authors conducted each interview, with an interpreter as required. Ten interviews were conducted with policymakers, academic and community-based researchers and research site staff and four were conducted with representatives of NGOs.

Interviews were audio recorded and transcribed. Thai language interviews were translated into English. Transcriptions were analyzed using both manual and computerized methods. Microsoft Word and Excel were used to code and sort text into themes [87]–[88]. The analysis was thematic and cumulative [89]. Themes identified as important within the GPP guidelines were sought and noted within the interview transcripts. These themes formed the basis of the analysis that follows.

Perspectives and recommendations were compared within and across stakeholder groups and analyzed for agreements and differences between groups. Here, owing to space limitations, perspectives are presented as overall reflections and recommendations for improving ethical and participatory practice for marginalized populations in biomedical HIV prevention trials.

## Results

Key informants provided input and recommendations for how to improve ethical and participatory practice for biomedical HIV prevention trials in Thailand. The focus was on three specific areas: standards of HIV prevention, informed consent and communication. Ethical practice and participatory practice were at times indistinguishable.

### Standards of HIV Prevention

Out of the discussions arose reflections on the normativity of ethics. From the perspectives of key informants, the very notion of ethics was seen as culturally dependent, embodying elements of morality and enforceability that varied with nation, community and institutional context:

*In terms of the ethics…some kind of knowledge [of ethics] is normal in the U.S. but sometime in Thailand people do not really know about this. Understandings of ethics in the U.S. and Thailand are different. [Academic Researcher]*

In spite of any internationality of ethical guidance, its role in the context of Thailand was not absolute. As expressed by an individual from the NGO sector who wondered about the applicability of ethical guidelines:

*OK. Belmont, Helsinki. GCP… are these ethical guidelines legally binding or are they morally binding. … They are what is called “good practice” … But are they enforceable?*

Even those key informants closest to the entities that develop, promote and review international ethical guidance and its application wondered about the sensibility of one-size-fits-all approaches to ethics in all communities. They wondered whether ethics like these should be tailored for the realities of different cultures and communities:

*How could we get an ethical and academic standard in research which makes sense?… but now again if it's a ‘we’ then who is ‘we’? It's finally the ethical commission in Thailand which endorses research or not and they have their own agenda and they have their own points of inference. (Policymaker)*

Guidance documents like GPP needed to be adaptable because populations targeted for recruitment to HIV prevention trials were diverse. Adaptability could ensure that community engagement activities to gain consensus on what services were delivered within a trial context would be flexible enough to serve diverse people's needs. However, even where international ethical guidelines were applied within prevention trial contexts, some international standards of prevention could be inadequate or non-existent in Thailand, depending on the population(s) in question.

Key informants from academic and NGO sectors suggested that despite international standards, attitudes towards prevention components for particular marginalized populations could influence the components of the prevention package able to be offered and delivered effectively. For marginalized populations such as sex workers, injecting drug users, and men who have sex with men, the absence of specific regulations, or the application of national standards that varied from international standards, could result in standards of prevention deemed unethical by international principles.

One example of how a lack of clarity regarding a best practice or standard of prevention could lead to confusion concerned the provision of clean injecting equipment for people who use drugs. While such a provision is indicated internationally as a needed and socially just component of a standard of prevention in a biomedical HIV prevention trial, in certain contexts in Thailand, such as when Thailand-based research may be U.S. funded, the provision of injecting equipment may not be possible. It was explained that this was due to U.S. policy perspectives concerning certain forms of harm reduction such as the provision of clean injecting equipment [1]. This could result in a disjuncture between what the community believed should and could be provided within a trial context under local laws, and what researchers and trial sponsors were willing to offer based on their interpretations of these local laws and policies, combined with any pressures applied by international donors. This conundrum persists despite international guidance or best practice.

Around such issues there was a quiet anger among advocates for injecting drug users who participated in this study's interviews. For these key informants, resolving the issue of differential interpretations relative to the ethics of standards of prevention that were possible and offered within a given trial, was identified as essential for future consideration.

Owing to an increasing importance within a prevention framework, marginalized populations like injecting drug users and sex workers were more visible within HIV prevention research contexts in Thailand. This could result in positive outcomes for the development of participatory ethics. Yet, the acceptability of marginalized populations within research contexts was not necessarily generalized outside of these specific research spheres, to the arena where some decisions about ethical and prevention practice were made. As one policymaker indicated, true participation at this level would most likely be attained when “non-threatening, non-violent, freedom of expression” was the norm. However, freedom of expression was not necessarily allowed or expected from all marginalized populations. The ability for community members and trial participants to speak out without fear to the people who conduct trials, and to have their concerns addressed was identified as another important issue for future consideration.

### Informed Consent

For key informants, ethical practice for informed consent was not universal but relative, variable and dependent on context. Key informants did not question informed consent as a cornerstone of ethics; but pointed to how, for the marginalized populations that make up the majority of those recruited into biomedical HIV prevention trials in Thailand, truly informed consent was contingent on a number of factors. Primary among these were elements of comprehension. It was recognized that it was one thing to read, write and communicate in a foreign language like English, but another thing to think about something as abstract as ethics in a way that diverged from cultural norms. Key informants felt that it was important for this potential variability at the level of cultural norms to be recognized by trial sponsors and implementers, the NGO sector and populations recruited. Concerning informed consent, one Community-based Researcher said:

*We [try to] make sure that they understand that their right is that they can stop during the study. But I'm pretty sure that less than 10% of them know that they can withdraw anytime. And because in Thai culture, when you sign something, you're obliged to the end.*

Innovations including the use of television, radio, social media, group training and one-on-one discussions could mitigate variability in knowledge, compression and cultural gap concerning informed consent, yet it could be challenging, because trial science and trial structure could be infused with different cultural and even disciplinary norms.

*I think [participants] have a glimpse but they don't understand the phase 2, phase 3, phase 4 of the trial…they don't know [these] procedures. [Policymaker]*

One approach to rectify this was to involve a Community Advisory Board (CAB) or other community advisory mechanism (CAM) to help work towards translating ethics for trial populations. A CAB or CAM could help in the development, delivery and refinement of the informed consent process. This reflected the importance of assessing both advisory members and study participants in terms of their comprehension of the informed consent process.

It was noted that in certain contexts in Thailand, an individual trial participant's ability to provide informed consent would be strengthened if her or his family and community had information about the decision the trial participant was facing. In Thailand, biomedical HIV prevention trials can target communities broadly—either via geographically-based communities or via communities of identity and association. Thus, providing community-level and community-wide education about incoming trials, potential recruitment processes, risks and benefits and other ethical considerations could be warranted. As a result, in addition to potential trial participants, it could be important within the context of a trial to communicate with community leaders, CABs, CAMs, and even family members, provided that individual autonomy was not compromised. Communicating trial specifics broadly could help to disseminate information about risks and benefits as well as information about prevention-related concerns such as adherence to pharmaceuticals, and possible side effects and their treatment.

Some researchers discussed how important it was for research staff to be well versed in all aspects of a study's protocol and to be able to translate this knowledge into plain language. It was suggested that this was particularly important for those who had direct contact with trial participants, such as nurses and counselors.

Some key informants described utilizing a CAB in the development and refinement of the informed consent process. This reflected the importance of assessing CAB members and study participants in terms of their comprehension of informed consent processes.

Key informants indicated that in Thailand, as in many places, trial participants were not particularly interested in reading long consent documents. This dislike could be exacerbated for those from marginalized groups for whom literacy and other barriers could be restraints. In practice, consent documents are often lengthy, owing to the requirements of international standards enforced by Institutional Review Boards (IRBs). Consent forms often use language that is very legalistic and challenging to understand. Further, poor translation to Thai from English could introduce additional challenges.

*First of all people don't like to read long documents. And consent forms are usually very long because you're required by the IRB to have all these sections and standard language that is very legal and difficult to understand… and translation from English to Thai, the sentence structures are different; it's the opposite. So it doesn't read well… It becomes very long and not very understandable. [Academic Researcher]*

*You might have an injecting drug user who has a little bit of withdrawal sitting in front of you, listening to you point out the consent process page by page. They won't be able to absorb all that much, maybe less than five per cent. [Academic Researcher]*

Echoing the academic researchers quoted above, some from the NGO sector spoke of the challenges participants could have in understanding that they were fully free to leave a study at any time. This freedom could be limited by cultural norms, hierarchical social structures and the dependence of individuals on access to health care services at point of recruitment. This spoke to the need to understand how to develop trials locally, which would be sensitive to culture so that participants would truly be able to cease their participation if that was their choice.

Key informants believed that despite the quality or complexity of an informed consent process, the reality was that for many from marginalized populations, money in the form of compensation for participation often was the primary motivating factor. Key informants from across stakeholder groups emphasized the harsh reality that if potential trial participants from marginalized populations were approached with a means to receive money, they frequently would not mind consenting, even without understanding the consent process or in some cases even reading the consent form. This was seen as especially true for people from the most marginalized groups. People might sign a long consent form without necessarily understanding fully the specifics of what it was they were providing consent for; and even more so in the case of a trial that offered a way to receive compensation on a regular basis, such as in the context of a clinical trial that occurred over a number of years.

### Communication

Throughout the research process, key informants repeatedly emphasized the act and role of communication as a means to improve ethical practice for marginalized populations in biomedical HIV prevention trials. At the same time, they indicated how challenging this could be, because often the language used in discussions of ethics and even some of the concepts underlying these ethics may not have local equivalents. Even when concepts could be considered universal, the language and jargon of science could challenge communication. As one Community-based Researcher indicated:

*There are lots of scientist vocabularies I can't understand. And when we sit down in a room and the researcher speaks, [it can be] very technical… So it's very high-level vocabularies and understanding. So the reaction… the communication should be improved to make people in the community think that even though they… have less education [and] don't know technical terms, they still can have some ability to respond… they still have some opportunity to learn… at the level that they can learn, so they can participate more.*

One NGO representative explained that communication issues were at the very core of what could challenge the development of ethical practice, particularly for marginalized populations. This was because community-based NGOs working on HIV issues among marginalized populations could themselves have limited understanding of trial science and processes. This could be made even more challenging when international research teams communicated in technical English. Such research jargon could complicate a participant's ability to comprehend the components required to participate freely.

Beyond the complexity of the language of research, some of the behaviours associated with marginalized populations like sex workers and injecting drug users could reveal the prejudices of researchers, trial staff and trial sponsors. Many key informants felt that effective communication could not occur in contexts where this kind of prejudice existed, or where mutual respect and mutual understanding were lacking or absent. One community advocate reflected the beliefs of many in suggesting that:

*First thing first, you must reduce your prejudice. This is just my thought. I think there were some prejudices that they had against each other among the community and the researchers. In this case, prejudice refers to the mistrust among each other. But if a mutual understanding could be established from the first place regarding the objective of the research, and if the research project saw the importance of the engagement, ethical issues, and the protection of the rights of the volunteers; the community and the researchers would be able to share a common goal. Then there would be a platform for reducing this prejudice from the beginning; and they would be able to carry out their work smoothly after that.*

For many informants, the importance of clear communication was fundamental. Such communication needed to be flexible enough to accommodate participants' questions, issues and complaints regardless of the social status of those that asked questions or voiced complaints. This reflected not only the challenge of universality from an ethical perspective but from a community point of view also. The notion of human rights was a clear illustration of this. Within some biomedical HIV prevention trial contexts in Thailand, human rights discourses could seem particularly Western and foreign to Thai culture. As one Academic Researcher explained:

*The way people may define rights may be different. In Thailand the principle of human rights may not be [taught in] general education…So people may see rights, the word ‘rights,’ in different way…So we may have different ways of interpreting this.*

For those from the community who were advocates or community-based researchers, and arguably closest to the experiences of participants from marginalized populations involved in biomedical HIV prevention trials, *ethical* prevention trials in the absence of human rights was not possible. In some ways, they recognized that human rights and ethics could be synonymous, yet such realization could be new and would require more consideration.

## Discussion

Our research has several limitations. The key informants who participated in interviews were selected based on their knowledge or experience. However, these key informants were not necessarily representative of all academic and community-based researchers, NGO representatives, research site staff, or policymakers. This research recruited more researchers than trial staff or representatives of NGOs. In addition, the majority of questions focused on the research enterprise as opposed to community development or community advocacy work. This may make it difficult for this research to reflect on the full breadth of experience.

Despite intentions of universality of ethical guidance, ethical practice was not a given. This was in part because it was not always normative or culturally appropriate to question the kind of majority action embodied within established forms of community participation as embedded within the machinery of biomedical HIV prevention trials. This was one reason so many indicated that CAMs and other similar mechanisms should begin as early as possible and should continue to be nurtured even after the end of a trial. This would not only promote consistency between trials but also would build on and solidify a knowledgeable, well-developed group to inform ongoing as well as future trials, sharing their expertise based upon a community-defined ethical perspective.

Key informants often came back to the theme of communication and to the importance of conveying sophisticated medical and research names and concepts in ways that could be understood by research participants. An important lesson regarding good communication was that it was a prerequisite, and that it formed the basis of trust between communities and trials.

For informants, trust would be required to improve ethical practice in biomedical HIV prevention trials, especially among those from socially marginal positions. Such inequities meant the roles traditional social order and power dynamics could play in the implementation and conduct of a prevention trial would need to be considered by trial teams. Power dynamics could influence informed consent processes as well as genuine input, for example the ability for participants to make complaints or to provide a research team with feedback.

This research points to ways in which the improvement of participatory practice within a biomedical HIV prevention trial would go hand in hand with improved ethical practice when involving marginalized populations in biomedical research. The improvement and maintenance of good ethical practice has been identified as an essential component of good participatory practice [14]-[15]. Owing to the susceptibility of many individuals from marginalized populations to the risks of HIV infection and transmission, the improvement of participatory practice within HIV prevention trials can not only improve ethical practice within these trials, but can help to reduce the risks of HIV infection and transmission within trial contexts and beyond.

The improvement of ethical research practice within initiatives like those undertaken as biomedical trials has been flagged as an important area for consultative research projects like ours to address [90]. This is particularly true in the context of biomedical HIV prevention trials where the optimism generated by the potential effectiveness of such trials may overshadow what Vural Ozdemir has described as IRB mission creep, ethical inflation and the underestimation of risks [91].

This research took place in Thailand and its findings apply primarily to Thailand [13], [63], [67]. However, the universality of many ethical considerations would suggest the results of this study can inform the improvement of ethical and participatory practice in other parts of the world where biomedical HIV prevention trials occur, and where clinical trials in marginalized populations continue.
